# A General Model of Negative Frequency Dependent Selection Explains Global Patterns of Human ABO Polymorphism

**DOI:** 10.1371/journal.pone.0125003

**Published:** 2015-05-06

**Authors:** Fernando A. Villanea, Kristin N. Safi, Jeremiah W. Busch

**Affiliations:** 1 School of Biological Sciences, Washington State University, PO Box 644236, Pullman, Washington, 99164, United States of America; 2 Department of Anthropology, Washington State University, PO Box 644910, Pullman, Washington, 99164, United States of America; University of Utah, UNITED STATES

## Abstract

The ABO locus in humans is characterized by elevated heterozygosity and very similar allele frequencies among populations scattered across the globe. Using knowledge of ABO protein function, we generated a simple model of asymmetric negative frequency dependent selection and genetic drift to explain the maintenance of ABO polymorphism and its loss in human populations. In our models, regardless of the strength of selection, models with large effective population sizes result in ABO allele frequencies that closely match those observed in most continental populations. Populations must be moderately small to fall out of equilibrium and lose either the A or B allele (*N_e_* ≤ 50) and much smaller (*N*_e_ ≤ 25) for the complete loss of diversity, which nearly always involved the fixation of the O allele. A pattern of low heterozygosity at the ABO locus where loss of polymorphism occurs in our model is consistent with small populations, such as Native American populations. This study provides a general evolutionary model to explain the observed global patterns of polymorphism at the ABO locus and the pattern of allele loss in small populations. Moreover, these results inform the range of population sizes associated with the recent human colonization of the Americas.

## Introduction

The maintenance of genetic variation has important consequences because heritable genetic variation fuels the evolutionary process. Balancing selection is of particular interest because it can produce stable genetic polymorphic systems [1]. Balancing selection serves as an umbrella term for several distinct processes (i.e., negative frequency dependent selection, heterozygote advantage, or fluctuating selection) that maintain higher than expected levels of heterozygosity and allelic diversity within populations. Examples of strong balancing selection are particularly compelling, as such a mode of selection can have profound impacts on patterns of genetic diversity across the genome [2]. In flowering plants and fungi, support for balancing selection has been taken from studies of single-locus self-incompatibility systems, whereby individuals sharing alleles at this locus cannot produce offspring [3,4,5]. Balancing selection has also been proposed to explain high genetic variability and the evolutionary stability of several polymorphisms in vertebrates, most notably the major incompatibility complex (MHC) [6,7,8], *opsin*, and the ABO blood groups of humans.

These three major polymorphisms in vertebrates are thought to be maintained through divergent mechanisms. MHC polymorphism has been associated with the fitness benefits of presenting a diverse array of antigens to recognize a rapidly evolving pathogen community. The high levels of MHC polymorphism have frequently been attributed to negative frequency dependent selection [7,8,9,10]. Balancing selection in the form of heterozygote advantage has been proposed to maintain multiple alleles of the X-linked color vision gene *opsin* in various New World monkey species [11,12,13]. However, studies of wild capuchin monkeys (*Cebus capucinus*) have failed to detect differences in caloric intake as a proxy for fitness between homozygous and heterozygous females during bouts of fruit foraging [14], and have found that heterozygous females are at a disadvantage when foraging for camouflaged insects [15]. Indeed, there has been relatively little direct support for the action of balancing selection on these two mammalian loci, although the maintenance of tremendous diversity and deep coalescence times between alleles at these loci provides indirect support for the action of balancing selection [7,13].

The ABO locus is one of the better studied genetic systems in humans. The ABO gene codes for a glycosyltransferase which modifies a precursor antigen into the A or B antigens. The O antigen results from a glycosyltransferase, which lacks enzymatic function. Human host antibodies are tolerant of self-produced ABO antigens, but agglutinate against foreign forms; thus, ABO phenotypes must be correctly identified to ensure successful blood transfusions. As a result of its medical importance, more is known about the geographic distribution of ABO alleles than for practically any other human biological trait [16,17,18]. Yet, in light of the medical importance of this genetic locus for tissue transplant, it is surprising how little attention has been focused on the evolutionary forces shaping the modern distribution of ABO alleles [18,19].

All primates share A and B blood groups, with evidence suggesting the polymorphism evolved very early in the ancestral primate, and has been maintained independently in multiple lineages for tens of millions of years through balancing selection [20]. Modern human alleles coalesce at around 2.6–2.5 mya [17,21,22], indicating that some of the ABO polymorphisms are unique to our species, particularly O alleles. The most elusive aspect of the genetic system is the long term maintenance of these O alleles. O alleles are typified by a deletion at position 261 of Exon 6, which creates a premature stop codon and results in a truncated protein lacking glycosyltransferase function [23]. In spite of lost enzymatic function, O alleles are apparently not deleterious, and have been maintained in human populations at relatively high frequencies. More surprisingly, O alleles are consistently found at higher frequencies than A or B alleles within modern human populations [24] and while they are functionally identical, currently there are over 40 O alleles that can be traced to at least five independent evolutionary origins in the distant past [16,25,26]. The maintenance of a class of O alleles with lost glycosyltransferase activity over such a long evolutionary time is consistent with a form of asymmetric negative frequency dependent selection (NFDS) where the O allele has some advantage over the A and B alleles. While other forms of selection could explain the observed patterns of polymorphism, such as diversifying selection or other forms of balancing selection, asymmetric NFDS offers a simpler explanation. A similar form of asymmetric NFDS is common in sporophytic self-incompatibility systems, where recessive S-alleles are subject to weaker NFDS compared to S-alleles with dominant expression [5,27,28].

**Fig 1:**
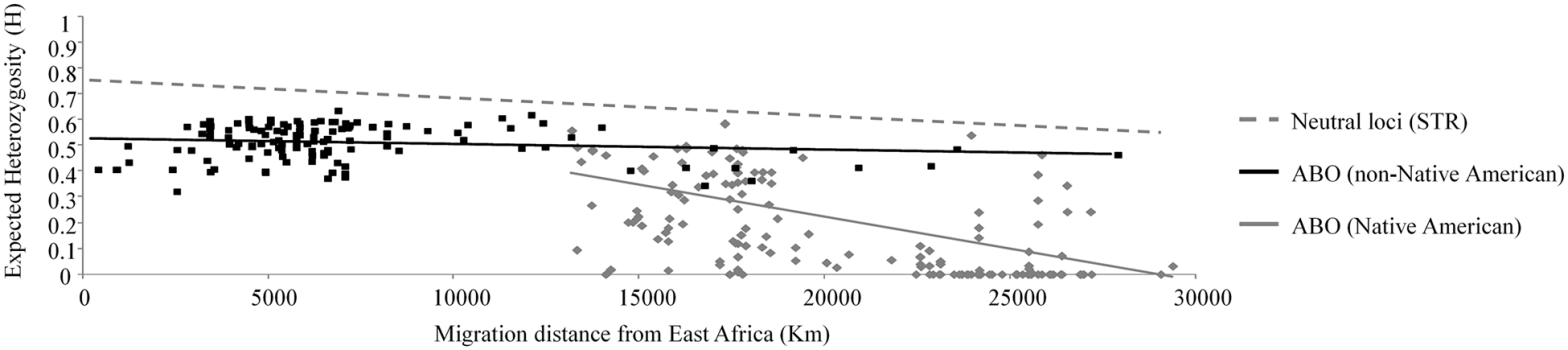
Pattern of isolation by distance at neutral markers and the ABO locus. The dashed grey line is based upon a regression analysis of heterozygosity at 678 autosomal short tandem repeats with migration distance from East Africa^43^. A similar regression was conducted using expected ABO heterozygosity, given allele frequencies at this locus (S2 Table). Native American populations (grey solid line) present a slope significantly different than zero (β = -2.532 x 10^–5^, *P*<<0.05), while non-Native American (black solid line) populations do not (β = -2.171 x 10^–6^, *P* = 0.0895).

The lack of a formal model to explain the distribution of human genetic variation at the ABO locus is driven by the poor understanding of the proximate functional mechanisms that provide the raw material for natural selection [23]. There is scarce evidence of immune function of the ABO locus, and in particular, include only a few reported examples of pathogen induced directional selection favoring A alleles [29] or B alleles [30] in isolated populations. These selective episodes could potentially account for locally high frequencies of a particular allele in some populations, but they cannot entirely explain the global pattern of polymorphism. Although there is currently no direct evidence supporting balancing selection, the relative uniformity of ABO allele frequencies across most human populations is consistent with its action (Fig 1). This is particularly remarkable given the wide geographical and ecological distribution of humans [22,23,31]. In addition to the relative constancy of this polymorphism across the globe, A, B, and O alleles are significantly older relative to segregating variation in neutral genomic regions with these alleles, exhibiting an approximately three-fold higher time to coalescence than expected under neutrality [21,22,32].

*Homo sapiens* is a species well known for exhibiting fairly small effective population sizes, particularly in isolated geographical contexts [33]. The effective population sizes of archaic populations, especially those that dispersed into previously uncolonized areas after the human diaspora out of Africa, were also likely to be very small [33,34], yet balancing selection on the ABO locus must have been strong enough to preserve variation in most cases. Two global populations, Native Americans and Native Australians, present an abnormally low diversity at the ABO locus. Native South American populations in particular are unique in that they have completely lost both the A and B alleles [18,24,35]. Understanding why the Native American pattern of diversity is distinct from other populations is crucial to ascertain whether or not balancing selection acts on this locus globally, and why its efficacy may have been attenuated in Native American populations.

In this study, we constructed a general model of negative frequency dependent selection (NFDS) which maintains a stable polymorphism of A, B, and O alleles during 100 human generations. We included stochastic fluctuations in allele frequency, whose magnitude was inversely proportional to the effective population size, *N*_*e*_ and we included reasonable strengths of selection. This model allowed us to estimate the range of effective population sizes in humans associated with the loss of allelic diversity. We then compared expectations taken from the model to observed patterns of ABO heterozygosity and variance among global populations, including Native Americans. This framework allowed three questions to be investigated: 1) Can asymmetric NDFS explain global patterns of polymorphism, even under weak selection?; 2) How small must *N*_*e*_ be to cause the loss of ABO polymorphism within human populations?; and 3) Is the loss of ABO polymorphism in small populations expected to be associated with biased fixation of the O allele, as has been observed in Native American populations? Evaluating these hypotheses permits progress to be made on a general evolutionary model that explains both the maintenance of ABO allele diversity across the globe and its loss in small populations.

## Results

### The model of NFDS at the ABO locus in finite human populations

**Fig 2:**
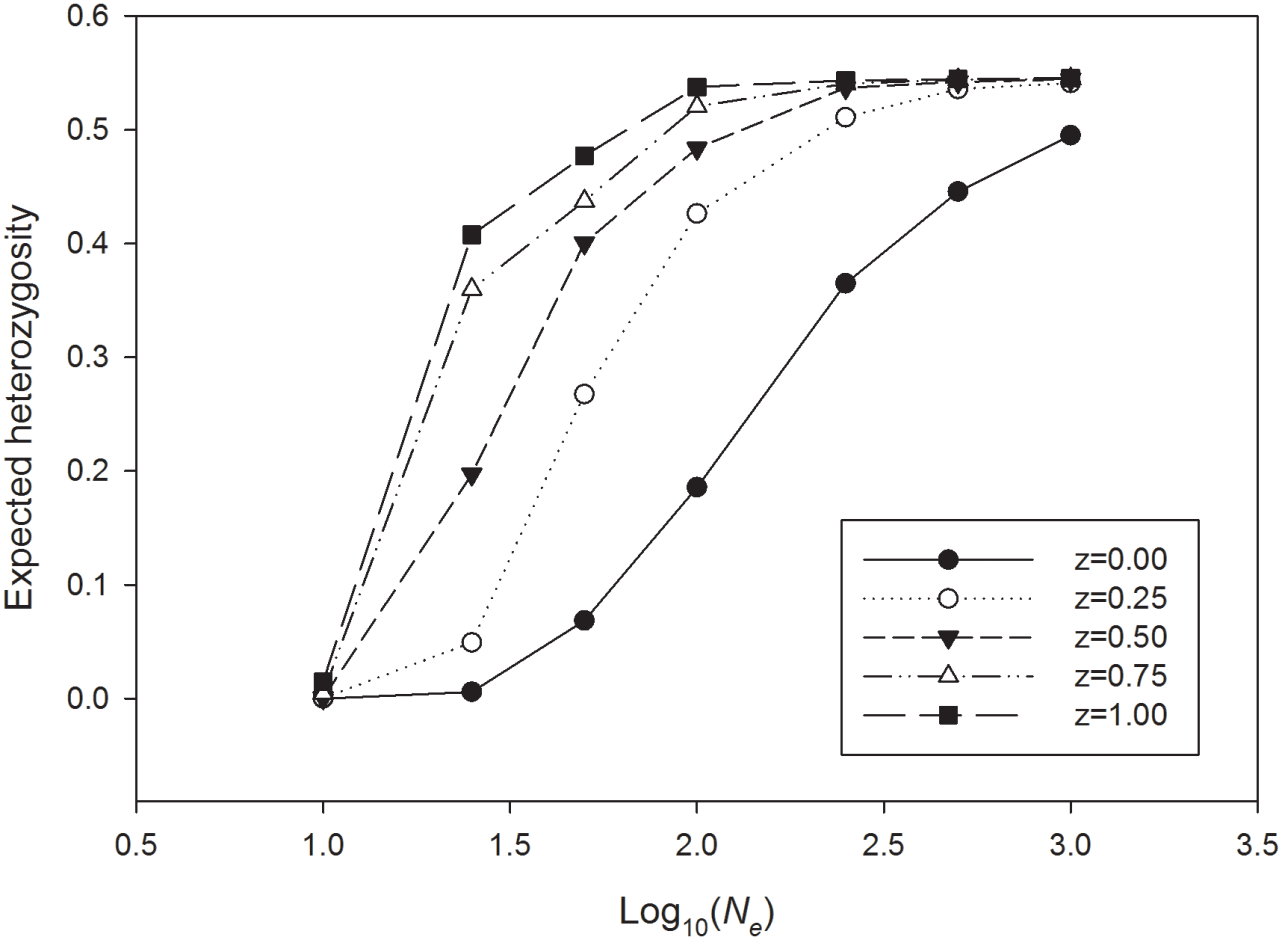
Reduction in ABO expected heterozygosity for different strengths of selection (*z* = 0.00 denotes neutrality). Results after 100 generations of selection and drift are shown across a range of log-transformed population sizes (*N*_*e*_). Simulations were conducted at untransformed *N*_*e*_ values of *N*_*e*_ = 10, 25, 50, 100, 250, 500, and 1,000.

NFDS at the ABO locus should be extremely robust in its ability to maintain variation if it is to explain patterns of polymorphism in most human populations. To verify that our approach correctly modeled the process of genetic drift, we examined the probability that the O allele fixed under complete neutrality (i.e. *z* = 0.0). We found that the probability of fixation for this allele was very close to its starting frequency of *r* = 0.62, as expected under neutrality (Table 1). The completely neutral case (*z* = 0.0) maintained much lower heterozygosity than seen in models that included natural selection. When natural selection is included, our model of ABO evolution maintained a higher equilibrium frequency of O alleles (*r*~0.62) than A or B alleles in large populations (i.e., *N*_*e*_ ≥100), regardless of the strength of selection, which is consistent with observations in modern populations (Table 1). For each non-zero strength of NFDS (*z*) studied in the model, expected heterozygosity maintained at the ABO locus within populations increased monotonically with *N*_*e*_, as expected if balancing selection counteracted genetic drift more effectively in large populations (Fig 2). Similar results were found in models with strong (*z* = 0.75) and very strong selection (*z* = 1.0) (Fig 2). A simple model-fitting analysis by least-squares between frequencies of the A, B, and O alleles in observed continental populations and the model outputs support a closer relation between non-zero strength models of NFDS than the neutral model (Table 2, S3 Table).

**Table 1:** The average frequency of the A, B, and O alleles ($\overset{-}{p}$, $\overset{-}{q}$, and $\overset{-}{r}$, respectively), the proportion of simulated populations in which alleles were lost, and the fraction of all simulations in which the O allele is fixed, for various strengths of selection (*z* = 1.00, 0.75, 0.5, 0.25, 0.00). 1,000 simulations were conducted at each effective population size (*N*_*e*_).

	*N*_*e*_	$\overset{-}{p}$	$\overset{-}{q}$	$\overset{-}{r}$	Proportion of simulations losing 1 allele	Proportion of simulations losing 2 alleles	Proportion of fixation events involving O allele
	1000	0.192	0.191	0.617	0.000	0.000	NA
	500	0.191	0.192	0.617	0.000	0.000	NA
	250	0.192	0.190	0.618	0.000	0.000	NA
(z) = 1.00	100	0.191	0.188	0.622	0.005	0.000	NA
	50	0.169	0.169	0.662	0.238	0.000	NA
	25	0.147	0.143	0.710	0.506	0.017	1.000
	10	0.011	0.012	0.977	0.983	0.965	0.988
	1000	0.192	0.191	0.617	0.000	0.000	NA
	500	0.192	0.191	0.617	0.000	0.000	NA
	250	0.191	0.190	0.620	0.000	0.000	NA
(z) = 0.75	100	0.186	0.183	0.632	0.051	0.000	NA
	50	0.152	0.160	0.688	0.381	0.000	NA
	25	0.125	0.137	0.739	0.557	0.115	0.983
	10	0.053	0.042	0.904	0.995	0.990	0.907
	1000	0.193	0.190	0.617	0.000	0.000	NA
	500	0.191	0.191	0.618	0.000	0.000	NA
	250	0.190	0.190	0.621	0.001	0.000	NA
(z) = 0.5	100	0.170	0.177	0.653	0.182	0.000	NA
	50	0.144	0.148	0.708	0.478	0.011	0.909
	25	0.107	0.103	0.790	0.748	0.496	0.883
	10	0.114	0.110	0.776	1.000	0.999	0.776
	1000	0.192	0.190	0.618	0.000	0.000	NA
	500	0.192	0.188	0.620	0.000	0.000	NA
	250	0.186	0.180	0.634	0.050	0.000	NA
(z) = 0.25	100	0.159	0.165	0.676	0.367	0.013	0.923
	50	0.134	0.140	0.726	0.641	0.297	0.805
	25	0.130	0.120	0.751	0.934	0.868	0.770
	10	0.145	0.178	0.677	1.000	0.999	0.677
	1000	0.195	0.192	0.613	0.018	0.000	NA
	500	0.183	0.197	0.620	0.133	0.011	1.000
	250	0.182	0.203	0.615	0.351	0.104	0.981
(z) = 0.00	100	0.169	0.198	0.633	0.711	0.481	0.786
	50	0.181	0.171	0.648	0.898	0.797	0.699
	25	0.179	0.188	0.633	0.990	0.980	0.637
	10	0.179	0.188	0.633	1.000	1.000	0.633

**Table 2:** Model fitting by least-squares [Σ(observed-expected)^2^].

Strength of selection	Model Fit
(z) = 1	0.680
(z) = 0.75	0.738
(z) = 0.5	0.847
(z) = 0.25	0.893
(z) = 0	1.092

Least-squares between frequencies of the A, B, and O alleles as an observed average in continental populations and the expected average in simulated populations (see S3 Table for details). Lower numbers indicate smaller differences between observed and expected frequencies.

### The probability and pattern of allele loss by genetic drift in the model

As modeled in this study, NFDS always maintains the A, B, and O alleles in populations with a minimum effective populations size of *N*_*e*_ = 250, even when the effect of selection is weak (*z* = 0.25), but if the strength of selection is strong, populations as small as *N*_*e*_ = 100 can maintain allele diversity (Table 1). As population sizes decrease below this number, the probability of losing an allele increases. Under weak selection (*z* = 0.25), when *N*_*e*_ = 100, any one allele is lost in 36.7% of all simulations, and when *N*_*e*_ ≤ 25, at least one allele is lost in all simulations (Table 1). The probability that all polymorphism is lost in the population is nonzero at sizes of *N*_*e*_ ≤ 50. When *N*_*e*_ = 25, complete loss of polymorphism occurs in 93.4% of all simulations, and in populations of size *N*_*e*_ < 10, fixation of a single allele is nearly always the only outcome (Table 1). As the strength of NFDS increases (*z* = 0.5, 0.75, 1.0), the effective population sizes required to maintain allele diversity decrease monotonically (Fig 2).

Because allele loss by means of pure genetic drift is random, the probability of fixation or loss of any given allele is a function of its starting frequency [1]. If the fixation of a single allele occurred multiple independent times, O alleles would have to become fixed every time, as no living population today is fixed for A or B alleles. Biased fixation of the O allele is achieved in our model through a mechanism of asymmetric balancing selection, where A and B alleles experience stronger NFDS, given their expression in both heterozygous and homozygous genotypes (see above definitions of fitness for genotypes). Given this asymmetric form of NFDS, the prevalence of the O allele increases as *N*_*e*_ declines, and this effect is pronounced in cases with strong selection (*z* = 0.75, 1.00). Such an effect causes O alleles to become fixed much more often than A or B alleles (Table 1) at low values of *N*_*e*_ where genetic drift overwhelms the capacity of NFDS to maintain polymorphism.

### The level and pattern of ABO heterozygosity in human populations

In comparison to the patterns of polymorphism at the ABO locus, neutral markers across the genome present a very characteristic geographic pattern; genetic diversity decreases as geographic distance increases from East Africa, consistent with serial bottlenecks as populations colonized new areas away from Africa [34,36]. In contrast, the ABO locus shows a different pattern (see Fig 1). The difference is particularly notable when Native American populations are analyzed separately from all other global populations, as the ABO locus in Native Americans only appears to behave in the same fashion as other neutral markers. This contrasting pattern of diversity implies recent serial bottlenecks in populations as humans colonized North America. While the reduced diversity is consistent with other autosomal markers, the number, degree, and magnitude of the putative bottlenecks have been strongly debated [36,37,38].

**Fig 3:**
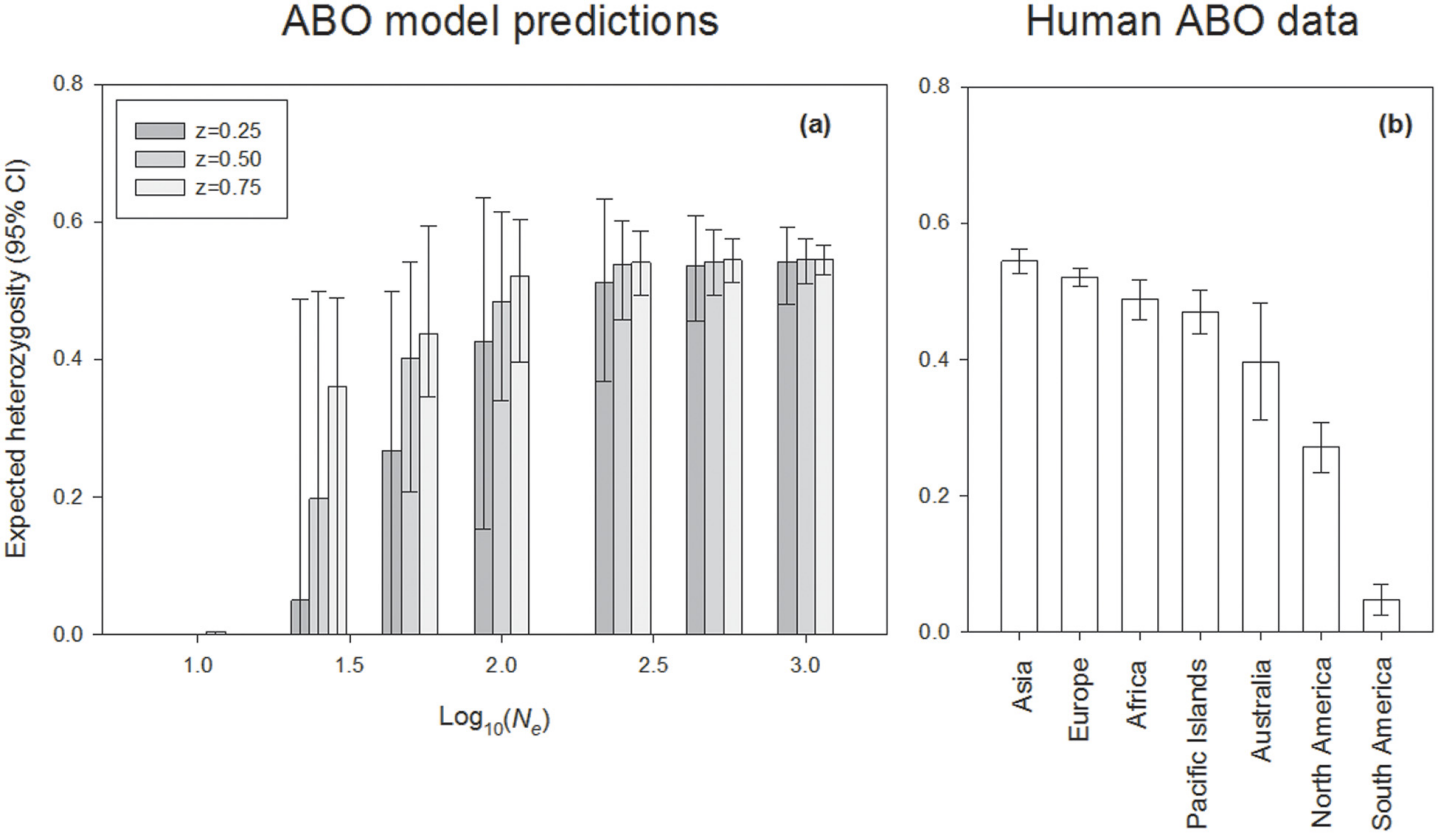
The relationship between ABO heterozygosity and log-transformed *N*_*e*_ based upon model expectations for weak selection (*z* = 0.25), moderate selection (*z* = 0.5) and strong selection (*z* = 0.75). Simulations were conducted at untransformed *N*_*e*_ values of *N*_*e*_ = 10, 25, 50, 100, 250, 500, and 1,000. Expected values of ABO heterozygosity are also shown for various non-Native American, North Native American, and South Native American populations.

Our model predicts that ABO heterozygosity should be relatively invariant in populations with *N*_*e*_ > 100, with a rapid decline and increase in variance below this threshold (Fig 3). Interestingly, levels of ABO heterozygosity and its variance in non-Native American populations fall within the 95% confidence interval for model predictions when *N*_*e*_ ≥ 100, except for cases of very strong selection (*z* = 1.00) and large effective size (*N*_*e*_ ≥ 500) (Table 1). ABO heterozygosity in modern Native American populations is reduced by more than half, falling within the broad 95% confidence interval of heterozygosity expected with weak selection when *N*_*e*_ = 100, weak to moderate selection when *N*_*e*_ = 50, or any strength of selection when *N*_*e*_ = 25 (Fig 3a and 3b). When Native Americans are divided into North and South American populations, there is significantly lower ABO heterozygosity in South America (Fig 3b). ABO heterozygosity in this region is consistent with a maximum *N*_*e*_ = 50 under weak selection (z = 0.25), or smaller populations (*N*_*e*_ = 25) with moderate or strong selection (z = 0.50, 0.75).

## Discussion

### A model of asymmetric NFDS compared to observed global patterns of polymorphism at the ABO locus

In relatively large populations, our simple general model of asymmetric NFDS predicts patterns of ABO polymorphism that are in close agreement with observations made in most human populations across the globe. Our model also predicts loss of ABO polymorphism and biased fixation of the O allele, which have been observed in Native Australians and Native Americans (Fig 3). The loss of allele diversity in these human populations has long been associated with low effective population sizes during the colonization of these continents, with the most extreme case in South America, where genetic drift should have the most pronounced influence.

The strength of selection in our model plays an important role in determining the long-term maintenance of ABO polymorphism. In particular, with increasingly strong natural selection, there are smaller threshold *N*_*e*_ values (Table 1) whereby ABO polymorphism is lost. Importantly, our models incorporating selection produce patterns of polymorphism that are consistent with empirical observations in human populations (Table 2). While the strength of balancing selection operating on ABO polymorphism is currently unknown, weak natural selection could arise via several potential sources, such as incomplete transmission of pathogens between hosts, additional components of the immune system influencing infection, or a delay between infection and mortality.

### The loss of ABO polymorphism and *N*_*e*_ in Native American populations

Native Americans differ genetically even from Asian populations such as Siberians, with whom they share relatively recent ancestry, yet the details concerning the timing and magnitude of the population bottleneck or bottlenecks associated with the colonization of the Americas are still unresolved [24,37,39]. Our model predicts populations must decline to a size of *N*_*e*_ ≤ 250 for the loss of either the A or B allele, as is the case with many North American populations, and populations must further decline to a size of *N*_*e*_ ≤ 50 for the loss of both of these alleles at the ABO locus, as is the case with South American populations. Since only the northernmost populations exhibit relatively high frequencies of the A and B alleles [40,41], these threshold *N*_*e*_ values are applicable to population structuring after the initial colonization, when various bands would have expanded and settled into North America. Alternatively, the frequency of A and B alleles in North America could have been increased by the more recent migrations, which contributed to the genetic make-up of Na-Dene and Aleut-Eskimo populations [42]. While this scenario is not mutually exclusive with localized demographic structuring diminishing variation, genetic contributions to other Native American groups outside Na-Dene have been calculated to be very limited [43,44].

Previous studies have estimated the effective population size of the ancestral Native American population based on genome-wide patterns of polymorphism analyzed in a neutral coalescent framework. These studies yield estimates as large as *N*_*e*_ = 1,500 for the ancestral Native American population [38,45]. Two models accounting for isolation with migration between Native American and Asian populations estimate a smaller effective population size in Native Americans (*N*_*e*_ = 87 [46] and *N*_*e*_ = 80 [47]), while another estimate based on autosomal diversity suggests effective population sizes of approximately *N*_*e*_ = 500, with a lower bound on the confidence of this estimate as low as *N*_*e*_ = 74 [48]. These variable estimates of effective population size are based on the coalescence of mitochondrial lineages in Asia at the time before the entrance into the continent, and thus reflect the effective population size of the entire Native American genealogy (reflecting the effective number of original migrants). These models are based on our understanding of the populations from which Native Americans descend, which most likely evolved in Siberia from 30,000 BP to 15,000 BP [37]. Our model provides a complementary line of evidence, reflecting demographic processes that need not be traced back to the original ancestral population. Instead, we propose the small effective population size required to explain the reduced allele diversity in most Native American populations is a reflection of the isolation of small demes that settled after they expanded further away from Siberia; the subsequent reduction could have occurred independently and multiple times, after populations were already established in the American continent.

The complete loss of both the A and B allele is extremely rare in populations globally, observed only in Central and South America, as modern populations in these regions are largely monomorphic for the O allele. ABO heterozygosity in these regions is consistent with a maximum possible *N*_*e*_ = 50 with weak NFDS (*z* = 0.25), reflecting the consequences of serial bottlenecks during the final phases of expansion throughout the Americas [34,36,38,49]. Importantly, these rather small values of *N*_*e*_ could have occurred infrequently during the colonization process. Since mutation to generate further A or B allele types from O alleles has never been reported, a single loss of polymorphism event in a settling population would entail reduced diversity at this locus for its descendant populations. Presumably, throughout this period of population structuring in the Americas, genetic drift has molded patterns of polymorphism throughout the genome of Native Americans [36,42,50].

While the predominance of the O allele in Native American populations long been appreciated [18,35,40,51], this pattern of diversity can provide novel insights into population history if ABO polymorphism is normally maintained by NFDS. Importantly, our simple evolutionary model also explains the predominance of the O allele in Native American populations. Specifically, the O allele is expected to stochastically fix within small populations because of its relatively high frequency, given an asymmetric selective advantage in comparison to the A and B alleles. In particular, A and B alleles are subject to much stronger negative selection when at higher frequencies. A similar form of asymmetric NFDS is common in sporophytic self-incompatibility systems, where recessive S-alleles are subject to weaker NFDS compared to S-alleles with dominant expression [27]. In these systems, recessive alleles reach higher frequencies and are shared more often between populations [5,28], such that recessive S-alleles would be the most likely to reach fixation if the ancestral mode of NFDS was suddenly weakened by effective neutrality in response to population bottlenecks. This breakdown of selective forces by the increased strength of genetic drift in Native American populations has been suggested in at least two other genetic systems [52,53].

Although the exact number of times the O allele was fixed throughout the Americas is of interest, our model provides little information toward answering this question. Since the model of ABO evolution predicts that the O allele should nearly always reach fixation in small populations, the predominance of this allele in many Native American populations is consistent with any number of fixation events. Nevertheless, the high among population variance in expected heterozygosity at the ABO locus among Native American populations is higher than observed outside of American populations, albeit lower than the variance generated by our model at low *N*_*e*_, where populations lost ABO diversity independently (Fig 3b). Outside of the ABO locus, relatively high among population variance has been reported in an analysis of 678 autosomal microsatellites (STR) across all human populations. In these analyses, Native Americans are consistently reported as the least heterozygous continental population, as well as the most highly structured [36]. Similarly, a model for the evolution of the Native American private allele D9S1120 [50] and the Native American private O allele O1vG542A [44] both emphasize high population structuring and the isolation of distinct groups after migration into the American continent. Isolation and structuring may have been facilitated by differences between emerging languages, and it should be noted that Native Americans possess the highest linguistic diversity of all continental populations, possibly the result of a high degree of isolation and structuring promptly following Native American dispersion [54].

### Alternative evolutionary processes associated with the collapse of ABO polymorphism

Several alternative evolutionary scenarios could also produce a pattern whereby ABO polymorphism would be lost in America yet retained elsewhere. Our model assumes that NFDS at the ABO locus acts in a similar manner across all human populations. Such an assumption is tenable considering the relatively constant frequencies of A, B, and O alleles that are observed in human populations, which are scattered across highly variable ecological settings in Africa, Europe and Asia, each of which presumably supports variable parasite communities. There is no known evidence suggesting this system would act differently on the American continent, although an altered selection regime providing a frequency-independent advantage to the O allele would be necessary to explain the biased fixation of this allele in Native American populations. Our model is not influenced by any prior information other than an assumption of NFDS that operates similarly in all populations. No assumptions were made about changes in the selection regime in America, and the inclusion of genetic drift is sufficient to explain the recurrent fixation of the O allele; we therefore conclude that bottlenecks associated with the colonization of America are, given our current understanding, a more parsimonious explanation of ABO polymorphism than alternative scenarios that invoke an environment-dependent selective advantage to the O allele.

It is also possible that the recent arrival of Europeans may have influenced patterns of ABO polymorphism in America. Admixture would have occurred in the decades following European colonization [55], and would have altered ABO frequencies in Native American populations; specifically, these events would be expected to re-introduce A and B alleles, which would enjoy a selective advantage when rare. Such a possibility is unlikely to be influential for several reasons. First, no ancient DNA study has ever found an allele, mitochondrial or nuclear, in pre-Colombian Native Americans which is not present in modern populations, suggesting that while populations were severely reduced by European influences, their genetic compositions remain similar [37,43,56,57,58,59]. Second, three independent ancient DNA studies have found similar ABO allele compositions in pre-Colombian Native American samples when compared to geographically close modern samples, suggesting the modern ABO geographic pattern reflects its pre-Colombian state [60,61,62]. Finally, if European admixture were occurring, one would expect a higher prevalence of ABO polymorphism in areas of Spanish conquest (i.e., southern North America and South America [63]), yet these regions are typified by the lowest occurrence of the A and B alleles in the New World.

## Conclusions

In this study, we present a simple general model to explain global patterns of polymorphism at the ABO locus by the interaction of balancing selection and genetic drift. In addition, we use this model to inform the evolutionary process through which the ancestral Native American population lost its diversity at this locus, standing apart from other global populations. The results of our simulations are in quantitative agreement with empirical data of ABO polymorphism. The agreement between empirical observations and model predictions provides insight into the demographic processes which reduced the effective population size of ancient Native American populations. In particular, our model supports historical periods with very small numbers (50 ≤ *N*_e_ ≤ 250) and is consistent with a period of more intense population structuring and isolation (25 ≤ *N*_*e*_ ≤ 50) in populations which colonized Central and South America.

## Methods

### Putative immune function of the ABO system

The physiological function of the ABO system in nature, outside of its role in compatible blood transfusions, remains poorly understood. ABO antigens are expressed in the erythrocytes and some epithelial cell membranes, where they form part of the glycocalyx. The glycocalyx is a negatively charged barrier which prevents spontaneous adhesion of red blood cells to themselves and to the endothelium, and putatively protects against pathogenic invasion [64]. Pathogens evolve much faster than long-lived vertebrates. Within a single host, an invading pathogen population should, in the absence of evolutionary constraints, adapt to overcome the host’s immune system, and to better invade a host’s cells. As pathogens invading new hosts with similar phenotypes would be better able to overcome the new host’s cell defenses, allelic diversity may therefore be maintained by NFDS [23]. The more limited structural function of the H antigen in O type hosts would cause asymmetric NFDS, as hosts with an O phenotype do not produce either A or B antigens.

### A model incorporating NFDS and genetic drift

We considered human populations where NFDS occurs in the zygote phase and was determined by ABO genotype [31]. We modeled ABO locus evolution as a deterministic outcome of natural selection. A previous model of ABO locus evolution by Seymour et al. [31] utilizes two alternating selection regimes exerted by “bacteria” or “viruses” to achieve equilibrium. In contrast, our model uses general equations for frequency dependent natural selection. Our model is built under the assumption that a human host generates antibodies that recognize foreign antigens. Fitness of an individual host phenotype depends on its ability to recognize pathogens based exclusively on the ABO antigen-antibody system. Pathogens which infected a host of a specific phenotype the previous generation are considered to have evolved to better infect hosts presenting the same cell membrane antigens; thus, the fitness of a host genotype is diminished by the presence of other host genotypes which express the same antigen phenotype. The fitness of particular host genotypes is a negative function of the frequency (*f*) of genotypes expressing the same antigen phenotype:

$$w_{AA}=1-zf_{AA}-zf_{AO}$$ (1.1)

$$w_{BB}=1-zf_{BB}-zf_{BO}$$ (1.2)

$$w_{BO}=1-zf_{BB}-zf_{BO}$$ (1.3)

$$w_{AB}=1-zf_{AB}-zf_{AA}-zf_{BB}-zf_{AO}-zf_{BO}$$ (1.4)

$$w_{OO}=1-zf_{OO}$$ (1.5)

The A, B, and O alleles of the ABO gene code for tranferases, which collectively determine a host phenotype by modifying the H antigen into A and B antigens, and in the instance of O, a defective tranferase which does not modify the H antigen. O heterozygotes are recessive and AB heterozygotes are co-dominant. Because the A and B alleles code for an enzyme with “trans” function, the A and B alleles are completely dominant over the O allele, as heterozygotes would still produce one functional copy of the glycosyltransferase enzyme, which would in turn convert all H antigens into A or B antigens [65]. Thus, homozygotes and heterozygotes for A or B phenotypes are treated equally in terms of fitness.

The strength of selection acting on the ABO locus should vary, depending upon the environment. We controlled the strength of selection exerted by pathogens in this model using a tuning parameter, (z) that ranges from 0 (neutrality) to 1 (very strong natural selection). We modeled ABO evolution across a range of selection intensity: z = 1, 0.75, 0.5, and 0.25. Importantly, we include the evolution of ABO allele frequencies when (z) = 0, a strictly neutral case. In particular, we verified that the probability that the O allele fixed under complete neutrality equaled the starting frequency of the allele [1]. In biological terms, (z) may account for incomplete transmission of pathogens between hosts, other components of the immune system overcoming infection, or a delay between infection and mortality. As an important note, the strength of selection z is not a selection coefficient (*s*) as understood in classic population genetics. There is no explicit equation relating (z) and (*s*), but instead, we have used the equations in the model to calculate a selection coefficient for each (z) value as the difference in absolute fitness between genotypes, where s is approximately one tenth of z (S1 Table); therefore, “strong” selection (z = 1.0) only corresponds to a selection coefficient of s = 0.10.

While O alleles always exhibit higher frequencies globally, A and B allele frequencies can vary among populations, most likely due to local directional selection pressures [29,30]. In particular, A alleles almost always exhibit higher frequencies than B alleles, but the evolutionary reasons (or proximal mechanisms) for this difference remain unclear. Because such localized selection regimes are beyond the scope of the model presented here, A and B allele initial frequencies and selection regimes are treated as equal in order to simplify the NFDS model, which is focused on understanding the fixation of O alleles. Change in allele frequency in response to NFDS was quantified using the relative fitness of genotypes:

$$p^{'}=\;\frac{p^{2}(w_{AA})+pq(w_{AB})+pr(w_{AO})}{\overset{-}{w}}$$ (1.6)

$$q^{'}=\;\frac{q^{2}(w_{BB})+pq(w_{AB})+qr(w_{BO})}{\overset{-}{w}}$$ (1.7)

$$r^{'}=\;\frac{r^{2}(w_{OO})+pr(w_{AO})+qr(w_{BO})}{\overset{-}{w}}$$ (1.8)

The initial frequencies of A, B, and O alleles (denoted as *p*, *q*, and *r*) took on starting values based on the model equilibrium frequencies (0.19, 0.19 and 0.62, respectively), which are simplified from the average global population frequencies (0.240, 0.133 and 0.627, respectively) when Native Americans and East Pacific populations are excluded. These populations have likely experienced strong genetic drift during their founding and are likely not in equilibrium (see S2 Table). We incorporated stochastic variation in allele frequencies in accordance with the Wright-Fisher model of genetic drift [1]. The frequency of alleles following selection (*p'*, *q'*, *r'*) were used as expected gamete frequencies, and a finite number of gametes equal to 2*N*_*e*_ were randomly sampled with replacement from this pool; random mating produced zygote frequencies. We varied the strength of genetic drift by running forward simulations in which *N*_*e*_ ranged from 10 to 10,000. Note that all results for *N*_*e*_ >1000 behave similarly, so we do not report larger values for simplicity. We measured the loss of diversity after 100 human generations, which corresponds to roughly 3,000 years (using 30 year generations [66]). We restricted the analysis to only 100 generations to reduce computational overhead, because the majority of allele loss events observed occur before 30 generations, and thus we believe we have captured the time frame over which allele loss is likely to occur.

Mutation from A/B alleles to O alleles was ignored in this model since the specific deletion at position 261 of Exon 6 is unlikely to appear in only 100 generations and reverse mutations from O alleles to A or B alleles should be unlikely.

All simulations were run in R for Mac OS X version 2.9.2 [67]. The resulting mean ABO allele frequencies were recorded after 1000 independent replicates of 100-generation cycles. The number of times one and two alleles were lost from populations were recorded. The expected heterozygosity (*H*_*e*_) and its variance at the ABO locus were calculated using *p*, *q*, and *r*; these values were compared with observations in human populations.

### Expected heterozygosity at the ABO locus

In order to generate estimates of ABO heterozygosity and variance in human populations across the globe, allele frequencies for 172 Native American and 137 non-Native American populations were calculated based on phenotypic blood type frequencies reported in literature (S2 Table) [61,68]. To reject a model of neutral evolution in favor of a model of NFDS, we compared the frequencies of the A, B, and O alleles averaged from these 209 extant populations with the frequencies from the model’s output, using least-squares. In addition to the pattern of allele loss in Native American populations, estimates of expected heterozygosity were compared to model results to infer the likely range of *N*_*e*_ values during and after the colonization of the American continent.

## Supporting Information

S1 Table Selection coefficient s calculated for each value of z. A selection coefficient was calculated as the difference in absolute fitness between genotypes, averaged over 100 generations. For each value of 'z', populations were simulated at allele equilibrium frequencies. In each generation, a selection coefficient was calculated for one chosen genotype using the reference equation: 's' = w(OO)—w(AA). A single genotype was chosen since the strength of selection increases uniformly as z approaches 1.0. The absolute difference between the fitness of the OO and AA genotypes each generation, averaged across all generations, was used to estimate s.
(DOCX)

S2 Table Empirical population data. Frequency of A, B, and O alleles at the ABO locus (p, q, and r), expected heterozygosity (He), and corrected geographic distance from East Africa for 172 Native American and 137 non-Native American populations (calculated using methods from Ramachandran et al. 2005).
(DOCX)

S3 TableModel fitting by least-squares [Σ(observed-expected)^2^].Least-squares between frequencies of the A, B, and O alleles ($\overset{-}{p}$, $\overset{-}{q}$, and $\overset{-}{r}$, respectively), as an average observed in continental populations and the average expected in simulated populations. Observed allele frequencies were first compared within regimes for each effective population size, and the best fit (yellow box) was added to calculate the absolute fit of each selection regime: a neutral model (z = 0), and four models of varying selection strength (*z* = 0.25, 0.5, 0.75, 1).(DOCX)

S1 File ABO Model script. R script of full model.
(TXT)
