# Supplementary material for: A General Model of Negative Frequency Dependent Selection Explains Global Patterns of Human ABO Polymorphism
Source: PLoS One. 2015 May 6;10(5):e0125003. doi: 10.1371/journal.pone.0125003 (PMC4422588; doi:10.1371/journal.pone.0125003)
Supplement: S1 Table — A selection coefficient was calculated as the difference in absolute fitness between genotypes, averaged over 100 generations. For each value of 'z', populations were simulated at allele equilibrium frequencies. In each generation, a selection coefficient was calculated for one chosen genotype using the reference equation: 's' = w(OO)—w(AA). A single genotype was chosen since the strength of selection increases uniformly as z approaches 1.0. The absolute difference between the fitness of the OO and AA genotypes each generation, averaged across all generations, was used to estimate s. (DOCX) [file pone.0125003.s001.docx]

**S1 Table.** Selection coefficient s calculated for each value of z. A selection coefficient was calculated as the difference in absolute fitness between genotypes, averaged over 100 generations. For each value of 'z', populations were simulated at allele equilibrium frequencies. In each generation, a selection coefficient was calculated for one chosen genotype using the reference equation: 's' = w(OO) - w(AA). A single genotype was chosen since the strength of selection increases uniformly as z approaches 1.0. The absolute difference between the fitness of the OO and AA genotypes each generation, averaged across all generations, was used to estimate s.

| Strength of selection (z) | Selection coefficient (s) |
| --- | --- |
| 1 | 0.093 |
| 0.75 | 0.073 |
| 0.50 | 0.049 |
| 0.25 | 0.025 |
| 0 | 0 |
