# Supplementary material for: A General Model of Negative Frequency Dependent Selection Explains Global Patterns of Human ABO Polymorphism
Source: PLoS One. 2015 May 6;10(5):e0125003. doi: 10.1371/journal.pone.0125003 (PMC4422588; doi:10.1371/journal.pone.0125003)
Supplement: S2 Table — Frequency of A, B, and O alleles at the ABO locus (p, q, and r), expected heterozygosity (He), and corrected geographic distance from East Africa for 172 Native American and 137 non-Native American populations (calculated using methods from Ramachandran et al. 2005). (DOCX) [file pone.0125003.s002.docx]

**S2 Table.** Frequency of A, B, and O alleles at the ABO locus (*p*, *q*, and *r*), expected heterozygosity (*H_e_)*, and corrected geographic distance from East Africa for 172 Native American and 137 non-Native American populations (calculated using methods from Ramachandran et al. 2005).

| Group | Population | *p* | *q* | *r* | *H_e_* | Distance (km) |
| --- | --- | --- | --- | --- | --- | --- |
| Native American | Aguaruna, Peru | 0.000 | 0.000 | 1.000 | 0.000 | 23697.025 |
| Native American | Algonkian | 0.266 | 0.016 | 0.716 | 0.416 | 17614.042 |
| Native American | Andes-Peru | 0.000 | 0.000 | 1.000 | 0.000 | 24435.205 |
| Native American | Apache | 0.413 | 0.000 | 0.587 | 0.485 | 17771.918 |
| Native American | Araucano (Mapuche), Argentina | 0.073 | 0.062 | 0.865 | 0.243 | 27171.452 |
| Native American | Arctic Village (Alaskan Indian) | 0.050 | 0.000 | 0.950 | 0.095 | 13315.486 |
| Native American | Assurini, Brazil | 0.000 | 0.000 | 1.000 | 0.000 | 25176.778 |
| Native American | Aweikoma, Brazil | 0.000 | 0.000 | 1.000 | 0.000 | 26885.374 |
| Native American | Aymara, Peru | 0.000 | 0.000 | 1.000 | 0.000 | 24150.882 |
| Native American | Aymara, Peru | 0.000 | 0.000 | 1.000 | 0.000 | 24150.882 |
| Native American | Bacairi, Brazil | 0.000 | 0.000 | 1.000 | 0.000 | 23862.165 |
| Native American | Beaver, Canada | 0.475 | 0.000 | 0.525 | 0.499 | 16257.445 |
| Native American | Bororo, Brazil | 0.000 | 0.000 | 1.000 | 0.000 | 25557.887 |
| Native American | Bororo-MatoGrosso | 0.000 | 0.000 | 1.000 | 0.000 | 26907.795 |
| Native American | Botocudo, Brazil | 0.000 | 0.000 | 1.000 | 0.000 | 27189.830 |
| Native American | Brazilian Amazon Indians | 0.000 | 0.000 | 1.000 | 0.000 | 24200.617 |
| Native American | Cacarapai, Brazil | 0.000 | 0.000 | 1.000 | 0.000 | 25186.506 |
| Native American | Caddo, Oklahoma | 0.056 | 0.000 | 0.944 | 0.106 | 18305.050 |
| Native American | Caddo, Oklahoma | 0.162 | 0.000 | 0.838 | 0.272 | 18493.576 |
| Native American | Caingang, Brazil | 0.000 | 0.000 | 1.000 | 0.000 | 26980.332 |
| Native American | Caingang, Brazil | 0.000 | 0.000 | 1.000 | 0.000 | 26980.332 |
| Native American | Caiua-MatoGrosso | 0.000 | 0.000 | 1.000 | 0.000 | 26355.879 |
| Native American | Calchaqui, Argentina | 0.263 | 0.000 | 0.737 | 0.388 | 25744.745 |
| Native American | Camayura, Brazil | 0.000 | 0.000 | 1.000 | 0.000 | 25156.820 |
| Native American | Camayura, Brazil | 0.000 | 0.000 | 1.000 | 0.000 | 25156.820 |
| Native American | Campa, Peru | 0.000 | 0.000 | 1.000 | 0.000 | 24185.335 |
| Native American | Cañari-Andes | 0.018 | 0.008 | 0.975 | 0.050 | 23117.838 |
| Native American | Cara-Ecuador | 0.019 | 0.007 | 0.975 | 0.050 | 23117.838 |
| Native American | Caraja, Brazil | 0.000 | 0.000 | 1.000 | 0.000 | 26059.809 |
| Native American | Catawba-SouthCarolina | 0.218 | 0.081 | 0.701 | 0.455 | 19401.113 |
| Native American | Cayapa, Ecuador | 0.000 | 0.016 | 0.983 | 0.033 | 22819.000 |
| Native American | Chaguanco+Chamaco+Chiriguano-Argentina | 0.000 | 0.000 | 1.000 | 0.000 | 25744.745 |
| Native American | Chanes, Argentina | 0.008 | 0.008 | 0.983 | 0.034 | 25501.505 |
| Native American | Cherokee, NC | 0.036 | 0.018 | 0.946 | 0.103 | 19210.875 |
| Native American | Cherokee-NorthCarolina | 0.018 | 0.009 | 0.973 | 0.053 | 19210.875 |
| Native American | Chipewayan, L. Athabasen | 0.100 | 0.100 | 0.800 | 0.340 | 16587.812 |
| Native American | Chippewa, Minnesota | 0.124 | 0.000 | 0.876 | 0.217 | 18725.991 |
| Native American | Chippewa-Minnesota | 0.064 | 0.000 | 0.936 | 0.120 | 17656.993 |
| Native American | Choctaw/Chickasaw-Oklahoma | 0.044 | 0.000 | 0.956 | 0.084 | 18522.695 |
| Native American | Choroti, Argentina | 0.000 | 0.000 | 1.000 | 0.000 | 25744.745 |
| Native American | Choroti, Argentina | 0.109 | 0.000 | 0.891 | 0.194 | 25744.745 |
| Native American | Chulupi, Argentina | 0.000 | 0.010 | 0.989 | 0.022 | 25501.505 |
| Native American | Chunupi, Argentina | 0.000 | 0.000 | 1.000 | 0.000 | 25501.505 |
| Native American | Chunupi, Argentina | 0.043 | 0.000 | 0.954 | 0.088 | 25501.505 |
| Native American | Colla, Argentina | 0.365 | 0.000 | 0.634 | 0.465 | 25853.169 |
| Native American | Colorado, Ecuador | 0.000 | 0.000 | 1.000 | 0.000 | 22822.563 |
| Native American | Cree Indians | 0.168 | 0.005 | 0.826 | 0.289 | 16210.655 |
| Native American | Cree, Alberta | 0.110 | 0.000 | 0.890 | 0.196 | 16167.386 |
| Native American | Cree-Edmonton, Montana/Alberta | 0.250 | 0.015 | 0.735 | 0.397 | 18103.922 |
| Native American | Dene-Canada | 0.112 | 0.010 | 0.878 | 0.216 | 14926.085 |
| Native American | Dieguenos, Southern California | 0.017 | 0.017 | 0.965 | 0.068 | 17656.238 |
| Native American | Dogrib, Mackenzie R. basin | 0.364 | 0.000 | 0.636 | 0.463 | 14094.117 |
| Native American | Fort Yukon (Alaskan Indian) | 0.160 | 0.000 | 0.840 | 0.269 | 13709.414 |
| Native American | Galibi, Brazil | 0.000 | 0.000 | 1.000 | 0.000 | 24507.348 |
| Native American | Guambino | 0.000 | 0.015 | 0.985 | 0.030 | 22589.744 |
| Native American | Guarani, Brazil | 0.000 | 0.000 | 1.000 | 0.000 | 25557.887 |
| Native American | Guarani, Brazil | 0.000 | 0.004 | 0.996 | 0.008 | 25557.887 |
| Native American | Guarani-MatoGrosso | 0.000 | 0.000 | 1.000 | 0.000 | 26159.033 |
| Native American | Haida | 0.104 | 0.009 | 0.887 | 0.202 | 14824.804 |
| Native American | Haida (Masset), North Is., Queen Charlottes | 0.250 | 0.025 | 0.725 | 0.411 | 15062.514 |
| Native American | Haida (Skidegate), South Is., Queen Charlottes | 0.098 | 0.007 | 0.895 | 0.189 | 15062.514 |
| Native American | Ho-Chunk-Nebraska | 0.238 | 0.000 | 0.762 | 0.363 | 17856.290 |
| Native American | Hopi | 0.065 | 0.000 | 0.935 | 0.122 | 17594.093 |
| Native American | Ica, Colombia | 0.026 | 0.009 | 0.965 | 0.068 | 22569.033 |
| Native American | Ingano | 0.009 | 0.048 | 0.942 | 0.110 | 22569.033 |
| Native American | Inuit-Aivilik-Canada | 0.425 | 0.000 | 0.575 | 0.489 | 16021.285 |
| Native American | Inuit-Angmagssalik, Greenland | 0.407 | 0.105 | 0.488 | 0.585 | 17308.103 |
| Native American | Inuit-Angmagssalik, Greenland | 0.439 | 0.103 | 0.458 | 0.587 | 17308.103 |
| Native American | Inuit-Augpilagtok, Greenland | 0.227 | 0.103 | 0.670 | 0.489 | 16263.776 |
| Native American | Inuit-CapeFarewell, Greenland | 0.331 | 0.025 | 0.644 | 0.475 | 17793.232 |
| Native American | Inuit-CapeYork, Greenland | 0.088 | 0.033 | 0.880 | 0.217 | 15808.943 |
| Native American | Inuit-Copper, Canada | 0.293 | 0.033 | 0.674 | 0.459 | 15374.656 |
| Native American | Inuit-Ittoqqortoormiit, Greenland | 0.356 | 0.014 | 0.630 | 0.476 | 16952.795 |
| Native American | Inuit-KodiakIsland, Alaska | 0.277 | 0.064 | 0.660 | 0.484 | 13731.174 |
| Native American | Inuit-KoniagIsolates, Alaska | 0.212 | 0.107 | 0.681 | 0.479 | 13731.174 |
| Native American | Inuit-Kuujjuaq, Canada | 0.211 | 0.013 | 0.776 | 0.353 | 17321.929 |
| Native American | Inuit-Labrador/Baffin Islands, Canada | 0.255 | 0.003 | 0.742 | 0.385 | 16796.587 |
| Native American | Inuit-Nome, Alaska | 0.259 | 0.074 | 0.667 | 0.483 | 12807.887 |
| Native American | Inuit-Thule, Greenland | 0.090 | 0.000 | 0.911 | 0.163 | 15706.829 |
| Native American | Inuit-Wainwright, Alaska | 0.358 | 0.090 | 0.552 | 0.559 | 13178.202 |
| Native American | Inupiat-PointBarrow, Alaska | 0.291 | 0.063 | 0.646 | 0.494 | 13329.007 |
| Native American | Isconahua, Peru | 0.000 | 0.000 | 1.000 | 0.000 | 24151.882 |
| Native American | Isconahua, Peru | 0.000 | 0.000 | 1.000 | 0.000 | 24150.882 |
| Native American | Jibaro-Amazonas | 0.000 | 0.000 | 1.000 | 0.000 | 23697.025 |
| Native American | Jivaro, Ecuador | 0.000 | 0.000 | 1.000 | 0.000 | 23697.025 |
| Native American | Kalapalo, Brazil | 0.000 | 0.000 | 1.000 | 0.000 | 25478.837 |
| Native American | Kutchin, Yukon | 0.000 | 0.009 | 0.991 | 0.018 | 14224.795 |
| Native American | Kwakiutl, British Columbia | 0.074 | 0.000 | 0.926 | 0.137 | 15492.450 |
| Native American | Kwakwaka'wakw+Nuu-chah-nulth +Haida+Salish | 0.065 | 0.003 | 0.931 | 0.128 | 15778.853 |
| Native American | Kwakwaka'wakw+Nuu-chah-nulth +Haida+Tlingit+Gitishan+Cowichan | 0.096 | 0.004 | 0.900 | 0.181 | 15778.853 |
| Native American | Loucheux, Mackenzie R. basin | 0.000 | 0.000 | 1.000 | 0.000 | 14094.117 |
| Native American | Macá-Paraguay | 0.000 | 0.000 | 1.000 | 0.000 | 26092.639 |
| Native American | Macu, Brazil | 0.000 | 0.000 | 1.000 | 0.000 | 24200.617 |
| Native American | Macuxi, Brazil | 0.004 | 0.004 | 0.992 | 0.016 | 24164.047 |
| Native American | Mapuche Indians, Chile | 0.136 | 0.069 | 0.795 | 0.345 | 26535.003 |
| Native American | Maricopa | 0.000 | 0.000 | 1.000 | 0.000 | 17776.331 |
| Native American | Mataco, Argentina | 0.000 | 0.000 | 1.000 | 0.000 | 26390.190 |
| Native American | Matacos, Argentina | 0.000 | 0.000 | 1.000 | 0.000 | 26392.190 |
| Native American | Matacos, Argentina | 0.037 | 0.000 | 0.963 | 0.071 | 26391.190 |
| Native American | Mato Grosso area, Brazil | 0.000 | 0.000 | 1.000 | 0.000 | 26907.795 |
| Native American | MatoGroso | 0.000 | 0.000 | 1.000 | 0.000 | 26907.795 |
| Native American | Maya, Yucatan | 0.009 | 0.005 | 0.987 | 0.027 | 20317.092 |
| Native American | Mehinaker, Brazil | 0.000 | 0.000 | 1.000 | 0.000 | 25156.820 |
| Native American | Mohave | 0.058 | 0.008 | 0.931 | 0.130 | 17521.194 |
| Native American | Muskhogean, Oklahoma | 0.086 | 0.000 | 0.914 | 0.157 | 19560.519 |
| Native American | Natives, Guatemala | 0.030 | 0.010 | 0.960 | 0.077 | 20646.987 |
| Native American | Navajo | 0.009 | 0.002 | 0.991 | 0.018 | 17648.741 |
| Native American | Navajo (Pinon, Arizona) | 0.270 | 0.000 | 0.730 | 0.394 | 17648.741 |
| Native American | Navajo, Arizona | 0.269 | 0.002 | 0.729 | 0.396 | 17650.741 |
| Native American | Navajo, New Mexico | 0.306 | 0.002 | 0.691 | 0.429 | 17648.496 |
| Native American | Navajo, New Mexico | 0.233 | 0.000 | 0.767 | 0.357 | 17647.496 |
| Native American | Navajo, Arizona | 0.146 | 0.002 | 0.852 | 0.254 | 17649.741 |
| Native American | Navajo, New Mexico | 0.005 | 0.000 | 0.996 | 0.009 | 17647.496 |
| Native American | Northern Athapaskan | 0.142 | 0.002 | 0.856 | 0.247 | 14926.071 |
| Native American | Nuu-chah-nulth | 0.008 | 0.000 | 0.992 | 0.015 | 15778.853 |
| Native American | Ojibwa, Manitoulin Island | 0.172 | 0.044 | 0.784 | 0.354 | 18561.177 |
| Native American | Ojibwa, Pikangikum | 0.225 | 0.000 | 0.775 | 0.349 | 17487.347 |
| Native American | Omaha, Nebraska | 0.056 | 0.003 | 0.941 | 0.111 | 17869.572 |
| Native American | Ona+Yamana+Alakaluf-Argentina | 0.000 | 0.000 | 1.000 | 0.000 | 29066.577 |
| Native American | Ona-Tierra del Fuego | 0.000 | 0.000 | 1.000 | 0.000 | 29066.577 |
| Native American | Paez, Colombia | 0.067 | 0.067 | 0.865 | 0.243 | 26537.749 |
| Native American | Palta, Ecuador | 0.012 | 0.008 | 0.980 | 0.040 | 23117.838 |
| Native American | Panzaleo, Ecuador | 0.021 | 0.008 | 0.972 | 0.056 | 21795.561 |
| Native American | Paramiteri, Brazil | 0.000 | 0.000 | 1.000 | 0.000 | 23117.838 |
| Native American | Pawnee, Oklahoma | 0.456 | 0.043 | 0.500 | 0.540 | 23946.152 |
| Native American | Pawnee,Oklahoma | 0.226 | 0.012 | 0.762 | 0.368 | 18300.366 |
| Native American | Penobscot, Maine | 0.270 | 0.002 | 0.728 | 0.397 | 18556.376 |
| Native American | Peru | 0.000 | 0.000 | 1.000 | 0.000 | 24150.882 |
| Native American | Peru | 0.016 | 0.059 | 0.924 | 0.142 | 24150.882 |
| Native American | Peru | 0.095 | 0.040 | 0.865 | 0.241 | 24150.882 |
| Native American | Piegans, Canada | 0.809 | 0.000 | 0.191 | 0.309 | 16059.930 |
| Native American | Pijao, Colombia | 0.000 | 0.000 | 1.000 | 0.000 | 22463.207 |
| Native American | Pillagas, Argentina | 0.174 | 0.000 | 0.826 | 0.287 | 25744.745 |
| Native American | Pima | 0.190 | 0.000 | 0.807 | 0.313 | 17759.608 |
| Native American | Piro, Peru | 0.000 | 0.000 | 1.000 | 0.000 | 25330.057 |
| Native American | Provine de Salta, Argentina | 0.000 | 0.000 | 1.000 | 0.000 | 25853.169 |
| Native American | Pueblo, New Mexico | 0.099 | 0.000 | 0.901 | 0.178 | 17862.701 |
| Native American | Quechua, Ecuador | 0.038 | 0.012 | 0.952 | 0.092 | 22819.000 |
| Native American | Quechua, Peru | 0.083 | 0.017 | 0.901 | 0.181 | 24150.882 |
| Native American | Salish, British Columbia | 0.200 | 0.000 | 0.800 | 0.320 | 15858.247 |
| Native American | Secoya, Ecuador | 0.000 | 0.000 | 1.000 | 0.000 | 22921.455 |
| Native American | Seminole-Florida | 0.019 | 0.004 | 0.977 | 0.045 | 20048.674 |
| Native American | Seminole-Oklahoma | 0.039 | 0.039 | 0.922 | 0.147 | 18418.104 |
| Native American | Shipibo, Peru | 0.000 | 0.000 | 1.000 | 0.000 | 23832.537 |
| Native American | Sioux (Pine Ridge, South Dakota) | 0.290 | 0.027 | 0.681 | 0.451 | 17431.419 |
| Native American | Sioux, South Dakota | 0.159 | 0.015 | 0.826 | 0.292 | 17431.419 |
| Native American | Stony-Morley, Montana/Alberta | 0.265 | 0.000 | 0.734 | 0.391 | 16985.992 |
| Native American | Swinomish, Washington/British Columbia | 0.188 | 0.027 | 0.785 | 0.348 | 15886.288 |
| Native American | Tariana+Tucano+Macu-Amazonas | 0.000 | 0.000 | 1.000 | 0.000 | 17431.419 |
| Native American | Tarina, Brazil | 0.000 | 0.000 | 1.000 | 0.000 | 17431.419 |
| Native American | Ticuna, Peru | 0.000 | 0.000 | 1.000 | 0.000 | 23619.404 |
| Native American | Tinglit, Alaska | 0.129 | 0.000 | 0.871 | 0.225 | 14971.275 |
| Native American | Tlingit, Alaska | 0.079 | 0.032 | 0.889 | 0.202 | 14696.154 |
| Native American | Toba, Argentina | 0.015 | 0.000 | 0.984 | 0.032 | 29386.609 |
| Native American | Toba, Argentina | 0.000 | 0.000 | 1.000 | 0.000 | 25501.505 |
| Native American | Tsimshian, British Columbia | 0.278 | 0.000 | 0.722 | 0.401 | 15114.423 |
| Native American | Tucano, Brazil | 0.000 | 0.000 | 1.000 | 0.000 | 23479.078 |
| Native American | Tunebo, Columbia | 0.010 | 0.010 | 0.980 | 0.039 | 22576.473 |
| Native American | Uaica, Brazil | 0.000 | 0.000 | 1.000 | 0.000 | 25569.522 |
| Native American | Uapixana, Brazil | 0.000 | 0.000 | 1.000 | 0.000 | 24129.336 |
| Native American | Unangan, Aleutian-Islands | 0.268 | 0.032 | 0.700 | 0.437 | 13430.125 |
| Native American | Uros, Peru | 0.000 | 0.000 | 1.000 | 0.000 | 24776.709 |
| Native American | Utes, Utah | 0.026 | 0.000 | 0.974 | 0.051 | 17169.797 |
| Native American | Utes, Utah | 0.019 | 0.000 | 0.981 | 0.037 | 17168.797 |
| Native American | Warrau, Venezuela | 0.000 | 0.000 | 1.000 | 0.000 | 23697.204 |
| Native American | Waura, Brazil | 0.000 | 0.000 | 1.000 | 0.000 | 25156.820 |
| Native American | Western Navajo | 0.425 | 0.000 | 0.575 | 0.489 | 17616.661 |
| Native American | Wichita, Oklahoma | 0.243 | 0.020 | 0.737 | 0.397 | 18322.326 |
| Native American | Xirixano, Brazil | 0.000 | 0.000 | 1.000 | 0.000 | 23946.152 |
| Native American | Yagua, Peru | 0.000 | 0.000 | 1.000 | 0.000 | 23079.875 |
| Native American | Yakima, Washington/British Columbia | 0.304 | 0.008 | 0.688 | 0.434 | 16147.537 |
| Native American | Yuma | 0.077 | 0.005 | 0.917 | 0.153 | 17746.409 |
| non-Native American | Ainu | 0.225 | 0.267 | 0.509 | 0.620 | 12090.333 |
| non-Native American | Algeria | 0.292 | 0.063 | 0.645 | 0.495 | 4125.434 |
| non-Native American | Angola | 0.186 | 0.133 | 0.681 | 0.484 | 2535.799 |
| non-Native American | Armenia | 0.379 | 0.109 | 0.512 | 0.583 | 4470.300 |
| non-Native American | Assam | 0.186 | 0.237 | 0.577 | 0.576 | 8715.417 |
| non-Native American | Australian Aborigines | 0.221 | 0.000 | 0.779 | 0.344 | 16763.418 |
| non-Native American | Austria | 0.276 | 0.097 | 0.627 | 0.521 | 5274.840 |
| non-Native American | Austria | 0.273 | 0.073 | 0.655 | 0.492 | 5274.840 |
| non-Native American | Bantu | 0.156 | 0.125 | 0.720 | 0.442 | 3342.951 |
| non-Native American | Beirut | 0.263 | 0.153 | 0.584 | 0.566 | 3410.032 |
| non-Native American | Belgium | 0.258 | 0.059 | 0.683 | 0.463 | 6175.015 |
| non-Native American | Bombay | 0.169 | 0.263 | 0.568 | 0.579 | 7185.202 |
| non-Native American | Borneo | 0.173 | 0.196 | 0.631 | 0.533 | 13164.371 |
| non-Native American | Bulgaria | 0.312 | 0.125 | 0.563 | 0.570 | 4485.651 |
| non-Native American | Central China | 0.218 | 0.210 | 0.572 | 0.581 | 10372.237 |
| non-Native American | Congo | 0.146 | 0.128 | 0.726 | 0.435 | 1233.315 |
| non-Native American | Cypriots | 0.308 | 0.104 | 0.588 | 0.548 | 3416.848 |
| non-Native American | Czechoslovakia | 0.285 | 0.148 | 0.568 | 0.575 | 5511.732 |
| non-Native American | Czechoslovakia | 0.255 | 0.150 | 0.595 | 0.558 | 5226.423 |
| non-Native American | Denmark | 0.283 | 0.080 | 0.637 | 0.508 | 6041.505 |
| non-Native American | Easter Island | 0.366 | 0.000 | 0.634 | 0.464 | 27910.217 |
| non-Native American | Egypt | 0.246 | 0.175 | 0.579 | 0.574 | 2800.395 |
| non-Native American | Eire | 0.198 | 0.070 | 0.732 | 0.420 | 7062.337 |
| non-Native American | Eire | 0.182 | 0.062 | 0.756 | 0.391 | 7062.337 |
| non-Native American | Eire | 0.168 | 0.064 | 0.768 | 0.378 | 7062.337 |
| non-Native American | England | 0.257 | 0.060 | 0.683 | 0.463 | 6492.816 |
| non-Native American | England | 0.267 | 0.060 | 0.673 | 0.472 | 6492.816 |
| non-Native American | Ethiopia | 0.155 | 0.096 | 0.748 | 0.407 | 915.825 |
| non-Native American | Europe | 0.280 | 0.132 | 0.588 | 0.558 | 6243.467 |
| non-Native American | Finland | 0.282 | 0.148 | 0.570 | 0.573 | 7171.444 |
| non-Native American | Finland | 0.287 | 0.124 | 0.589 | 0.556 | 6726.928 |
| non-Native American | France | 0.280 | 0.060 | 0.661 | 0.482 | 6594.249 |
| non-Native American | France | 0.290 | 0.049 | 0.661 | 0.477 | 6593.249 |
| non-Native American | France | 0.293 | 0.053 | 0.654 | 0.484 | 6592.249 |
| non-Native American | France | 0.221 | 0.019 | 0.760 | 0.373 | 6591.249 |
| non-Native American | France | 0.313 | 0.080 | 0.607 | 0.527 | 6590.249 |
| non-Native American | France | 0.254 | 0.051 | 0.695 | 0.450 | 4470.300 |
| non-Native American | French West Africa | 0.142 | 0.152 | 0.706 | 0.458 | 5376.979 |
| non-Native American | Georgia | 0.330 | 0.108 | 0.562 | 0.563 | 4597.933 |
| non-Native American | Germany | 0.272 | 0.119 | 0.608 | 0.542 | 5752.884 |
| non-Native American | Germany | 0.285 | 0.101 | 0.614 | 0.532 | 5751.884 |
| non-Native American | Germany | 0.284 | 0.112 | 0.604 | 0.542 | 5750.884 |
| non-Native American | Germany | 0.281 | 0.091 | 0.627 | 0.519 | 5749.884 |
| non-Native American | Germany | 0.282 | 0.076 | 0.642 | 0.502 | 5748.884 |
| non-Native American | Germany | 0.288 | 0.076 | 0.636 | 0.507 | 5747.884 |
| non-Native American | Germany | 0.285 | 0.070 | 0.645 | 0.498 | 5746.884 |
| non-Native American | Ghana | 0.130 | 0.122 | 0.748 | 0.408 | 3531.386 |
| non-Native American | Greece | 0.248 | 0.094 | 0.658 | 0.496 | 4553.830 |
| non-Native American | Greece | 0.248 | 0.098 | 0.655 | 0.500 | 4553.830 |
| non-Native American | Hawaii | 0.378 | 0.013 | 0.608 | 0.487 | 23564.725 |
| non-Native American | Hindus | 0.174 | 0.246 | 0.579 | 0.573 | 7802.600 |
| non-Native American | Hindus | 0.224 | 0.218 | 0.557 | 0.591 | 7381.710 |
| non-Native American | Hungary | 0.322 | 0.143 | 0.535 | 0.590 | 5053.398 |
| non-Native American | Hungary | 0.202 | 0.259 | 0.539 | 0.601 | 5053.398 |
| non-Native American | Iceland | 0.192 | 0.070 | 0.738 | 0.413 | 17582.960 |
| non-Native American | Israel | 0.273 | 0.124 | 0.603 | 0.546 | 3199.854 |
| non-Native American | Italy | 0.248 | 0.102 | 0.650 | 0.506 | 5351.265 |
| non-Native American | Italy | 0.251 | 0.080 | 0.669 | 0.483 | 5350.265 |
| non-Native American | Italy | 0.275 | 0.076 | 0.649 | 0.497 | 5349.265 |
| non-Native American | Japan | 0.266 | 0.177 | 0.558 | 0.587 | 12402.054 |
| non-Native American | Java | 0.227 | 0.188 | 0.584 | 0.571 | 13987.262 |
| non-Native American | Jugoslavia | 0.167 | 0.175 | 0.658 | 0.508 | 4801.378 |
| non-Native American | Kazakhstan | 0.255 | 0.269 | 0.476 | 0.636 | 6873.424 |
| non-Native American | Kenya | 0.162 | 0.090 | 0.748 | 0.406 | 893.344 |
| non-Native American | Khorezm | 0.249 | 0.204 | 0.548 | 0.597 | 5770.216 |
| non-Native American | Korea | 0.237 | 0.231 | 0.532 | 0.607 | 11298.082 |
| non-Native American | Kurds | 0.253 | 0.187 | 0.560 | 0.588 | 4158.001 |
| non-Native American | Lebanon | 0.356 | 0.136 | 0.508 | 0.597 | 3410.032 |
| non-Native American | Lebanon | 0.291 | 0.104 | 0.605 | 0.538 | 3410.032 |
| non-Native American | Libya | 0.190 | 0.128 | 0.683 | 0.482 | 2916.435 |
| non-Native American | Madagascar | 0.202 | 0.230 | 0.568 | 0.584 | 3239.130 |
| non-Native American | Madras | 0.158 | 0.231 | 0.612 | 0.548 | 8191.069 |
| non-Native American | Marathas | 0.191 | 0.163 | 0.646 | 0.520 | 7185.335 |
| non-Native American | Micronesia | 0.216 | 0.114 | 0.670 | 0.491 | 17001.139 |
| non-Native American | Moluccas | 0.124 | 0.124 | 0.752 | 0.403 | 14757.991 |
| non-Native American | Mongols | 0.201 | 0.196 | 0.603 | 0.558 | 9290.176 |
| non-Native American | Morocco | 0.194 | 0.156 | 0.650 | 0.515 | 5126.513 |
| non-Native American | Moscow | 0.260 | 0.162 | 0.578 | 0.572 | 5839.877 |
| non-Native American | Moslems | 0.197 | 0.242 | 0.562 | 0.587 | 7073.579 |
| non-Native American | Mozambique | 0.141 | 0.103 | 0.756 | 0.398 | 3430.284 |
| non-Native American | N Indians | 0.182 | 0.230 | 0.587 | 0.569 | 7137.737 |
| non-Native American | Nepal | 0.245 | 0.190 | 0.565 | 0.585 | 8224.954 |
| non-Native American | Netherlands | 0.270 | 0.064 | 0.666 | 0.479 | 6168.105 |
| non-Native American | Netherlands | 0.246 | 0.048 | 0.706 | 0.439 | 6167.105 |
| non-Native American | Netherlands | 0.266 | 0.061 | 0.673 | 0.473 | 6166.105 |
| non-Native American | New Guinea | 0.143 | 0.114 | 0.743 | 0.415 | 16253.451 |
| non-Native American | New Guinea | 0.143 | 0.114 | 0.743 | 0.415 | 20910.997 |
| non-Native American | New Zealand | 0.213 | 0.059 | 0.728 | 0.421 | 22865.099 |
| non-Native American | Nigeria | 0.127 | 0.124 | 0.749 | 0.407 | 2414.504 |
| non-Native American | North Australia | 0.184 | 0.041 | 0.776 | 0.363 | 18025.758 |
| non-Native American | Norway | 0.327 | 0.062 | 0.611 | 0.516 | 6476.577 |
| non-Native American | Omsk | 0.271 | 0.156 | 0.573 | 0.574 | 7146.838 |
| non-Native American | Pakistan | 0.191 | 0.257 | 0.552 | 0.593 | 6781.818 |
| non-Native American | Persia | 0.225 | 0.157 | 0.618 | 0.543 | 4907.737 |
| non-Native American | Philippines | 0.168 | 0.160 | 0.672 | 0.495 | 12467.045 |
| non-Native American | Poland | 0.285 | 0.123 | 0.593 | 0.553 | 5411.517 |
| non-Native American | Poland | 0.264 | 0.173 | 0.563 | 0.584 | 5410.517 |
| non-Native American | Portugal | 0.297 | 0.053 | 0.650 | 0.487 | 7218.574 |
| non-Native American | Rhodesia | 0.088 | 0.099 | 0.813 | 0.322 | 2529.274 |
| non-Native American | Romania | 0.335 | 0.148 | 0.517 | 0.598 | 4465.337 |
| non-Native American | Romania | 0.365 | 0.142 | 0.493 | 0.604 | 4464.337 |
| non-Native American | Samarkand | 0.174 | 0.276 | 0.550 | 0.591 | 6231.361 |
| non-Native American | Scotland | 0.210 | 0.071 | 0.719 | 0.434 | 6856.678 |
| non-Native American | Senegal | 0.134 | 0.141 | 0.725 | 0.437 | 5475.371 |
| non-Native American | Sierra Leone | 0.159 | 0.151 | 0.691 | 0.475 | 4970.998 |
| non-Native American | Sikhs | 0.188 | 0.221 | 0.591 | 0.566 | 6970.991 |
| non-Native American | Sinhalese | 0.146 | 0.168 | 0.685 | 0.481 | 8511.035 |
| non-Native American | South Africa | 0.199 | 0.142 | 0.659 | 0.506 | 3942.865 |
| non-Native American | South Australia | 0.591 | 0.000 | 0.409 | 0.483 | 19145.578 |
| non-Native American | Spain | 0.365 | 0.127 | 0.508 | 0.593 | 6716.117 |
| non-Native American | Spain | 0.231 | 0.027 | 0.742 | 0.395 | 6715.117 |
| non-Native American | Spain | 0.286 | 0.050 | 0.664 | 0.475 | 6590.249 |
| non-Native American | Sudan | 0.170 | 0.163 | 0.668 | 0.499 | 1204.727 |
| non-Native American | Sumatra | 0.126 | 0.200 | 0.674 | 0.490 | 11823.974 |
| non-Native American | Sweden | 0.306 | 0.075 | 0.619 | 0.518 | 6211.007 |
| non-Native American | Sweden | 0.421 | 0.043 | 0.536 | 0.534 | 6211.007 |
| non-Native American | Switzerland | 0.273 | 0.073 | 0.654 | 0.492 | 5754.156 |
| non-Native American | Switzerland | 0.315 | 0.060 | 0.625 | 0.506 | 5754.156 |
| non-Native American | Syria | 0.365 | 0.107 | 0.528 | 0.576 | 3435.446 |
| non-Native American | Tadzhik | 0.218 | 0.233 | 0.549 | 0.597 | 6403.804 |
| non-Native American | Taiwan | 0.215 | 0.196 | 0.588 | 0.569 | 11525.053 |
| non-Native American | Tamils | 0.144 | 0.230 | 0.626 | 0.535 | 8190.743 |
| non-Native American | Tamils | 0.150 | 0.182 | 0.668 | 0.498 | 8190.743 |
| non-Native American | Thailand | 0.161 | 0.231 | 0.608 | 0.551 | 10098.999 |
| non-Native American | Tunisia | 0.232 | 0.117 | 0.651 | 0.509 | 4178.560 |
| non-Native American | Turkey | 0.322 | 0.109 | 0.569 | 0.561 | 3916.836 |
| non-Native American | Turkey | 0.362 | 0.062 | 0.576 | 0.533 | 3915.836 |
| non-Native American | Uganda | 0.138 | 0.113 | 0.749 | 0.407 | 406.994 |
| non-Native American | Ukraine | 0.276 | 0.152 | 0.572 | 0.574 | 4998.723 |
| non-Native American | USSR | 0.227 | 0.154 | 0.619 | 0.542 | 5837.877 |
| non-Native American | USSR | 0.251 | 0.187 | 0.562 | 0.586 | 5836.877 |
| non-Native American | USSR | 0.279 | 0.134 | 0.587 | 0.559 | 5835.877 |
| non-Native American | USSR | 0.272 | 0.126 | 0.602 | 0.548 | 5834.877 |
| non-Native American | Vietnam | 0.176 | 0.181 | 0.643 | 0.523 | 10258.958 |
| non-Native American | Yemen | 0.185 | 0.062 | 0.752 | 0.396 | 4917.323 |
| non-Native American | Yemen | 0.151 | 0.094 | 0.754 | 0.399 | 4916.323 |
| non-Native American | Yugoslavia | 0.293 | 0.124 | 0.583 | 0.559 | 4801.378 |
