# Supplementary material for: A General Model of Negative Frequency Dependent Selection Explains Global Patterns of Human ABO Polymorphism
Source: PLoS One. 2015 May 6;10(5):e0125003. doi: 10.1371/journal.pone.0125003 (PMC4422588; doi:10.1371/journal.pone.0125003)
Supplement: S3 Table — Least-squares between frequencies of the A, B, and O alleles (p-, q-, and r-, respectively), as an average observed in continental populations and the average expected in simulated populations. Observed allele frequencies were first compared within regimes for each effective population size, and the best fit (yellow box) was added to calculate the absolute fit of each selection regime: a neutral model (z = 0), and four models of varying selection strength (z = 0.25, 0.5, 0.75, 1). (DOCX) [file pone.0125003.s003.docx]

**S3 Table.** Model fitting by least-squares [Σ(observed-expected)^2^] between frequencies of the A , B, and O alleles ($\bar{p},\bar{q},\mathrm{and} \bar{r},$respectively), as an average observed in continental populations and the average expected in simulated populations. Observed allele frequencies were first compared within regimes for each effective population size, and the best fit (yellow box) was added to calculate the absolute fit of each selection regime: a neutral model (z= 0), and four models of varying selection strength (*z*= 0.25, 0.5, 0.75, 1).

| Observed frequencies |  | *p* | *q* | *r* |
| --- | --- | --- | --- | --- |
| South America | | 0.025 | 0.006 | 0.969 |
| North America | | 0.178 | 0.017 | 0.805 |
| Australia |  | 0.332 | 0.014 | 0.655 |
| Pacific Islands | | 0.207 | 0.117 | 0.676 |
| Africa |  | 0.208 | 0.128 | 0.664 |
| Europe |  | 0.272 | 0.107 | 0.621 |
| Asia |  | 0.219 | 0.182 | 0.599 |

| Expected frequencies | *N_e_* | *p* | *q* | *r* | South America | North America | Australia | Pacific Islands | Africa | Europe | Asia | Absolute Model Fit |
| --- | --- | --- | --- | --- | --- | --- | --- | --- | --- | --- | --- | --- |
|  | 1000 | 0.195 | 0.192 | 0.613 | 0.436 | 0.261 | 0.229 | 0.099 | 0.083 | 0.114 | 0.029 |  |
|  | 500 | 0.183 | 0.197 | 0.620 | 0.428 | 0.259 | 0.239 | 0.101 | 0.086 | 0.126 | 0.044 |  |
|  | 250 | 0.182 | 0.203 | 0.615 | 0.434 | 0.266 | 0.245 | 0.109 | 0.094 | 0.132 | 0.046 |  |
| (z) = 0 | 100 | 0.169 | 0.198 | 0.633 | 0.413 | 0.250 | 0.247 | 0.100 | 0.086 | 0.137 | 0.062 | 1.092 |
|  | 50 | 0.181 | 0.171 | 0.648 | 0.393 | 0.220 | 0.218 | 0.066 | 0.053 | 0.114 | 0.063 |  |
|  | 25 | 0.179 | 0.188 | 0.633 | 0.412 | 0.242 | 0.233 | 0.088 | 0.073 | 0.123 | 0.053 |  |
|  | 10 | 0.179 | 0.188 | 0.633 | 0.412 | 0.243 | 0.233 | 0.088 | 0.074 | 0.123 | 0.053 |  |

| Expected frequencies | *N_e_* | *p* | *q* | *r* | South America | North America | Australia | Pacific Islands | Africa | Europe | Asia | Absolute Model Fit |
| --- | --- | --- | --- | --- | --- | --- | --- | --- | --- | --- | --- | --- |
|  | 1000 | 0.192 | 0.190 | 0.618 | 0.430 | 0.255 | 0.228 | 0.095 | 0.079 | 0.115 | 0.034 |  |
|  | 500 | 0.192 | 0.188 | 0.620 | 0.428 | 0.253 | 0.227 | 0.093 | 0.077 | 0.114 | 0.035 |  |
|  | 250 | 0.186 | 0.180 | 0.634 | 0.410 | 0.237 | 0.222 | 0.079 | 0.064 | 0.113 | 0.048 |  |
| (z) = 0.25 | 100 | 0.159 | 0.165 | 0.676 | 0.359 | 0.197 | 0.230 | 0.068 | 0.062 | 0.138 | 0.099 | 0.893 |
|  | 50 | 0.134 | 0.140 | 0.726 | 0.298 | 0.152 | 0.245 | 0.092 | 0.097 | 0.176 | 0.159 |  |
|  | 25 | 0.130 | 0.120 | 0.751 | 0.268 | 0.126 | 0.248 | 0.107 | 0.116 | 0.193 | 0.186 |  |
|  | 10 | 0.145 | 0.178 | 0.677 | 0.360 | 0.209 | 0.250 | 0.087 | 0.081 | 0.155 | 0.107 |  |
|  |  |  |  |  |  |  |  |  |  |  |  |  |
|  |  |  |  |  |  |  |  |  |  |  |  |  |

| Expected frequencies | *N_e_* | *p* | *q* | *r* | South America | North America | Australia | Pacific Islands | Africa | Europe | Asia | Absolute Model Fit |
| --- | --- | --- | --- | --- | --- | --- | --- | --- | --- | --- | --- | --- |
|  | 1000 | 0.193 | 0.190 | 0.617 | 0.431 | 0.256 | 0.228 | 0.096 | 0.080 | 0.115 | 0.033 |  |
|  | 500 | 0.191 | 0.191 | 0.618 | 0.430 | 0.256 | 0.229 | 0.096 | 0.080 | 0.116 | 0.035 |  |
|  | 250 | 0.190 | 0.190 | 0.621 | 0.427 | 0.253 | 0.229 | 0.094 | 0.078 | 0.116 | 0.037 |  |
| (z) = 0.5 | 100 | 0.170 | 0.177 | 0.653 | 0.387 | 0.221 | 0.231 | 0.075 | 0.063 | 0.128 | 0.073 | 0.847 |
|  | 50 | 0.144 | 0.148 | 0.708 | 0.320 | 0.166 | 0.237 | 0.077 | 0.080 | 0.160 | 0.137 |  |
|  | 25 | 0.107 | 0.103 | 0.790 | 0.219 | 0.112 | 0.277 | 0.152 | 0.163 | 0.236 | 0.235 |  |
|  | 10 | 0.114 | 0.110 | 0.776 | 0.237 | 0.117 | 0.267 | 0.136 | 0.146 | 0.221 | 0.217 |  |

| Expected frequencies | *N_e_* | *p* | *q* | *r* | South America | North America | Australia | Pacific Islands | Africa | Europe | Asia | Absolute Model Fit |
| --- | --- | --- | --- | --- | --- | --- | --- | --- | --- | --- | --- | --- |
|  | 1000 | 0.192 | 0.191 | 0.617 | 0.431 | 0.256 | 0.229 | 0.096 | 0.080 | 0.115 | 0.034 |  |
|  | 500 | 0.192 | 0.191 | 0.617 | 0.431 | 0.256 | 0.229 | 0.096 | 0.080 | 0.116 | 0.034 |  |
|  | 250 | 0.191 | 0.190 | 0.620 | 0.428 | 0.254 | 0.229 | 0.094 | 0.078 | 0.116 | 0.036 |  |
| (z) = 0.75 | 100 | 0.186 | 0.183 | 0.632 | 0.413 | 0.240 | 0.225 | 0.082 | 0.067 | 0.115 | 0.046 | 0.738 |
|  | 50 | 0.152 | 0.160 | 0.688 | 0.344 | 0.186 | 0.234 | 0.071 | 0.068 | 0.147 | 0.113 |  |
|  | 25 | 0.125 | 0.137 | 0.739 | 0.283 | 0.147 | 0.255 | 0.105 | 0.112 | 0.191 | 0.174 |  |
|  | 10 | 0.053 | 0.042 | 0.904 | 0.080 | 0.161 | 0.375 | 0.285 | 0.298 | 0.364 | 0.374 |  |

| Expected frequencies | *N_e_* | 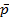   \| *p* \| \| --- \| | 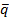   \| *q* \| \| --- \| | 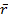   \| *r* \| \| --- \| | South America | North America | Australia | Pacific Islands | Africa | Europe | Asia | Absolute Model Fit |
| --- | --- | --- | --- | --- | --- | --- | --- | --- | --- | --- | --- | --- | --- | --- | --- |
|  | 1000 | 0.192 | 0.191 | 0.617 | 0.431 | 0.256 | 0.229 | 0.096 | 0.080 | 0.115 | 0.033 |  |
|  | 500 | 0.191 | 0.192 | 0.617 | 0.431 | 0.257 | 0.230 | 0.097 | 0.081 | 0.117 | 0.035 |  |
|  | 250 | 0.192 | 0.190 | 0.618 | 0.430 | 0.255 | 0.228 | 0.095 | 0.079 | 0.115 | 0.034 |  |
| (z) = 1 | 100 | 0.191 | 0.188 | 0.622 | 0.425 | 0.251 | 0.227 | 0.091 | 0.075 | 0.114 | 0.037 | 0.680 |
|  | 50 | 0.169 | 0.169 | 0.662 | 0.376 | 0.209 | 0.225 | 0.066 | 0.056 | 0.126 | 0.081 |  |
|  | 25 | 0.147 | 0.143 | 0.710 | 0.317 | 0.160 | 0.232 | 0.074 | 0.077 | 0.158 | 0.138 |  |
|  | 10 | 0.011 | 0.012 | 0.977 | 0.017 | 0.240 | 0.455 | 0.374 | 0.387 | 0.452 | 0.464 |  |
